# Salivary cortisol in post-traumatic stress disorder: a systematic review and meta-analysis

**DOI:** 10.1186/s12888-018-1910-9

**Published:** 2018-10-05

**Authors:** Xiongfeng Pan, Zhipeng Wang, Xiaoli Wu, Shi Wu Wen, Aizhong Liu

**Affiliations:** 1 Department of Epidemiology and Health Statistics, Xiangya School of Public Health, Central South University, Changsha, China; 2 Department of Obstetrics and Gynaecology, University of Ottawa, Ottawa, ON Canada; 3 Ottawa Hospital Research Institute, Ottawa, ON Canada

**Keywords:** Post-traumatic stress disorder, Salivary cortisol, Systematic review, Meta-analysis

## Abstract

**Background:**

Studies investigating salivary cortisol level as susceptibility marker for post-traumatic stress disorder (PTSD) produced inconsistent results. The aim of this study was to compare salivary cortisol concentration levels in PTSD patients with those in controls by synthesizing published data.

**Methods:**

We did a systematic review, meta-analysis and meta-regression of studies comparing concentrations of salivary cortisol between patients with PTSD and controls. The electronic databases of PubMed, Embase, Web of Science and Psyc-ARTICLES were searched for relevant articles. A random-effects model with restricted maximum-likelihood estimator is used to synthesize the effect size (assessed by standardized mean difference).

**Results:**

A total of 784 articles were identified of which 22 were included in the final analysis. A trend of lower salivary cortisol levels was found in PTSD patients when compared with the controls (SMD = − 0.28, 95% CI-0.53;-0.04, *p* = 0.022). Subgroup analysis showed that the salivary cortisol levels were lower in patients with PTSD than in controls in studies conducted after 2007 or in studies using saliva samples collected in the morning.

**Conclusions:**

The evidence from this meta-analysis supports that salivary samples collected in the morning consistently showed a lower salivary cortisol level in patients with PTSD than in controls, although whether salivary cortisol could be used as a diagnostic tool requires further research.

**Electronic supplementary material:**

The online version of this article (10.1186/s12888-018-1910-9) contains supplementary material, which is available to authorized users.

## Background

Post-traumatic stress disorder (PTSD) is a common psychiatric and anxiety disorder caused by traumatic events [1]. It has a negative impact on the physical and mental health of the affected patients [2], as well as their professional and social life [3, 4], which may impose a large burden on patients’ families and society. Indeed, PTSD is a kind of complex disease which may also be related to genetic factors (internal factors) and environmental factors (external factors) [5, 6]. Its etiology and pathogenesis have not been fully understood [6]. However, there is plenty of evidence that PTSD might be attributed to the disorder of the Hypothalamic-Pituitary-Adrenal axis (HPA axis) [7, 8]. Cortisol is an adrenal glucocorticoid hormones secreted by the zona fasciculata in the adrenal cortex [9, 10]. It is the end product of the HPA axis in humans. When faced with stressors the body may produce a corresponding stress response [11]. At the same time the body may secrete large amounts of cortisol to inhibit stress response by metabolic action, which in turn restores the body back to its normal functionality [12, 13]. However, if the body has been in a state of high pressure which stimulates its stress response too often, then this may lead to passivation of the HPA axis [10, 14]. Moreover, if the HPA axis is not restored to normal, then abnormal cortisol levels may arise in patients with PTSD [15]. Therefore, cortisol could be used as a biomarker for patients with PTSD [16].

Various biological specimens, including plasma, serum, saliva, cerebrospinal fluid and urine are used to measure cortisol [12, 17]. In addition, acquisition process of salivary measurement has been proposed as a noninvasive method [18]. We focus on the salivary cortisol mainly for the consideration of practice applications. Salivary cortisol is more readily available than urine specimens, and is more convenient to collect at home for large scale epidemiological studies [19].

A much debated question is whether salivary cortisol could be used as a susceptibility marker for PTSD patients [20]. Studies investigating salivary cortisol as a susceptibility marker for PTSD patients have produced different results [12], which may be attributed to the differences in investigation time, sampling time, type of trauma, assessment tools for PTSD symptoms, and collection and analysis of salivary cortisol [21, 22].

There has been no meta-analysis specifically targeted on salivary cortisol as a susceptibility marker for PTSD patients, although some previous studies have included salivary cortisol in subgroup analyses [12].

The purpose of this study is to compare salivary cortisol levels between PTSD patients and controls using a meta-analysis of existing studies. We also used regression and subgroup analyses to explore the sources of heterogeneity among studies [23].

## Methods

### Identification and selection of studies

**Fig. 1:**
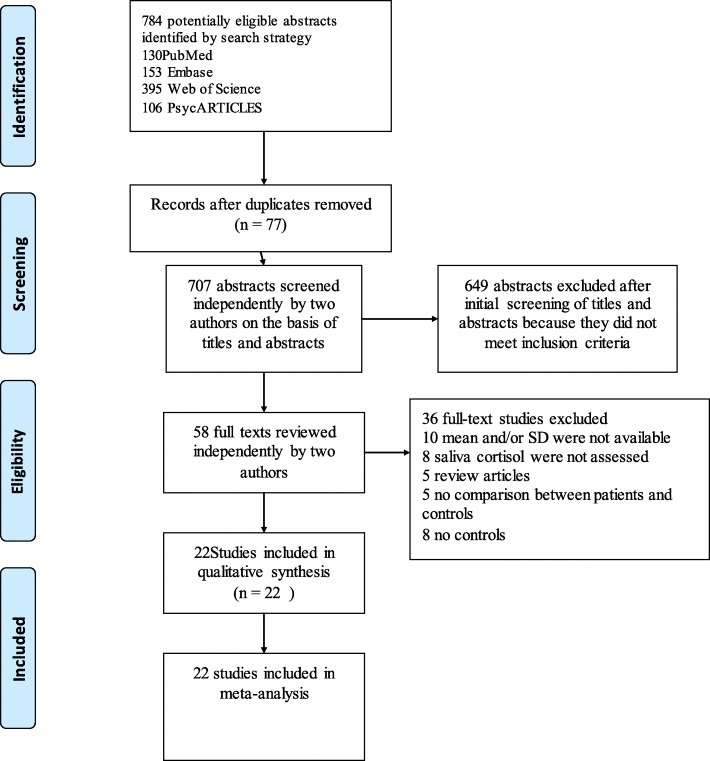
Study selection

PRISMA guidelines were used for this systematic review and meta-analysis [24]. Online electronic databases were searched from September 1987 until September 2017 for articles published in English. These included PubMed, Embase, Web of Science and Psyc-ARTICLES. Experienced librarians designed these searches, which used the following keywords:(((((((((((cortisol in saliva [Title/Abstract]) OR saliva cortisol [Title/Abstract]) OR glucocorticoids in saliva[Title/Abstract]) OR saliva glucocorticoid[Title/Abstract])OR steroid hormones in saliva [Title/Abstract]) OR saliva steroid [Title/Abstract]) OR corticosteroids in saliva[Title/Abstract]) OR saliva corticosteroid[Title/Abstract])) OR Salivary Cortisol[Title/Abstract]))AND((PTSD[Title/Abstract]) OR Posttraumatic Stress Disorder[Title/Abstract]). These terms were adapted for the other databases and the detailed search strategies are shown in the Additional file 1. The detailed search strategies are shown in the Fig. 1.

### Eligibility criteria

Two researchers independently screened and selected the relevant articles. In case of disagreement the final decision was made after consultation with a third party [25]. Moreover, the grey literatures were not included in our study. Primary inclusion criteria for articles for this study were: the study included PTSD cases and control groups, reported PTSD diagnostic criteria, and reported the mean and standard deviation (SD) of salivary cortisol. Exclusion criteria were: studies investigating PTSD with other comorbid disorders, or HPA axis disorder and review articles [26].

### Data extraction

This study used Note Express of Central South University in order to gain insights into analyses, and manage articles. Trauma-exposed controls (TC) and non-trauma-exposed controls (NTC) were all eligible as control groups. Since most studies reported TC for 81.8% (18/22), we chose TC as control groups when both TC and NTC were included. In addition, we analyzed the relation between PTSD and all NTC as control groups as a supplement [27]. The following characteristics were extracted from each eligible article: first author, publication year, sex, ages of participants, participant number, salivary cortisol concentration level mean ± standard deviation, country where the study was conducted (study country), trauma type [28], PTSD assessment method, saliva collection time [28], salivary cortisol collection and assay methods, inter-assay variation, intra-assay variation, sensitivity and T-frozen (temperature of frozen). Salivary cortisol unit conversion which we used was 1 g/dl = 27.59 nmol/L [29].

### Statistical analyses

All analyses were carried out using R software (version R i386 3.4.2). Meta-analysis and meta-regression analysis were performed using R software with package metafor [30]. The random-effects model with restricted maximum-likelihood estimator was used to synthesize the effect size in the studies. Standardised mean difference (SMD) was used to assess the effect size, calculated by using Cohen’s d. Significance level was set at 0.05 for all statistical tests. If the SMD level was ≤0.2 then it was considered low effect; if it was 0.2–0.7 then moderate effect; if it was ≥0.8 then large effect [31, 32]. Begg’s rank correlation test was used to check publication bias. The Q statistic was used to test the presence of heterogeneity and the I^2^ statistic was used to quantify the percentage of variability. Maximal heterogeneity is indicated by an I^2^ = 100% whereas no heterogeneity is indicated by an I^2^ = 0 [33]. In addition, subgroup analyses were conducted with regard to saliva collection time and study year. We also performed meta-regression analysis to explore other sources of heterogeneity. The following 8 variables were included in this analysis: Country (USA = 1,other = 0), PTSD assessment (DSM-IV CAPS = 1, other = 0), collection time (am = 1,pm = 0), publication year (study year≥2007 = 1, < 2007 = 0), assayed methods (report = 1,Unreported = 0), inter-assay variation (report = 1,Unreported = 0), intra-assay variation (report = 1,Unreported = 0), sensitivity (report = 1,Unreported = 0) and frozen (report = 1,Unreported = 0).

## Results

### Literature search

Literature search produced an aggregate of 784 relevant articles of which 130 were from PubMed, 153 from Embase, 395 from Web of Science and 106 from PsycARTICLES. Of these, 77 were excluded because they were duplicates. Further assessment of abstracts of the 707 remaining articles resulted in 649 exclusions for failing to meet the inclusion criteria. The 58 full articles left were reviewed by two authors independently. Then 10 articles were excluded for not reporting means (SDs), 8 articles were excluded for not assessing saliva cortisol, 5 articles were excluded because they were review articles, 5 articles were excluded for not reporting results comparing patients and controls, and 8 articles were excluded for not reporting results in controls. In the end, 22 eligible articles were included for this study (Fig. 1).

### Characteristics of eligible articles

Table 1 presents characteristics of the 22 eligible studies. Most articles reported saliva collection time, salivary cortisol collection and assay methods, inter-assay variation, intra-assay variation, sensitivity and T-frozen.

**Table 1:** Characteristics of studies included in the meta-analysis

Study	N	Country	Trauma type	Controls	Female	Mean Age	PTSD Assessment	Collection time	Assayed Methods	Interassay variation	Intra-assay variation	Sensitivity	T-frozen
Carrion 2001 [43]	51	USA	Mixed trauma	NTC	21	10.7	DSM-IV Reaction Index	AM,PM	RIA	12%	NR	NR	-20 °C
Coupland 2003 [44]	66	Canada	Abuse	NTC	66	38 ± 11	DSM-IV CAPS-1	AM,PM	ELISA	2%	8%	0.05 mg/dl.	−80 °C
Feldman 2013 [45]	232	Israeli	Combat	TC,NTC	110	1.5–5.0	Clinicians diagnose	AM	ELISA	10.5%	13.4%	NR	−20 °C
Gill 2008 [46]	71	USA	Civilian trauma	TC,NTC	71	42.9 ± 7.82	DSM-IV CAPS	PM	ELISA	9%	9%	NR	−80 °C
Kloet 2006 [47]	83	Netherland	Combat	TC,NTC	0	34.1 ± 5.8	DSM-IV CAPS	AM,PM	RIA	4%	5.5–9%	NR	−80 °C
Kobayashi 2014 [48]	39	USA	Injury	TC	7	40.3 ± 10.7	DSM-IV CAPS	AM,PM	RIA	NR	NR	NR	−20 °C
Lindauer 2006 [49]	24	Netherland	Mixed trauma	TC	10	35.1 ± 11.4	DSM-IV CAPS	AM,PM	RIA	10%	10%	NR	−20 °C
Lipschitz, DS 2003 [50]	48	USA	Mixed trauma	TC,NTC	37	16.4 ± 2.6	DSM-IV CTQ	AM	RIA	8.00%	9.00%	NR	−80 °C
Mcfarlane 2011 [51]	48	Australia	Traumatic accident	TC	12	34 ± 12.7	DSM-IV CAPS	AM,PM	RIA	NR	NR	NR	− 20 °C
Neylan 2009 [52]	22	USA	Combat	TC	0	51.1 ± 2.5	DSM-IV SCID	AM	NR	NR	NR	NR	NR
Tucker 2010 [53]	100	USA	Bombing registry	TC,NTC	54	47.0 ± 10.0	DSM-IV DIS	AM	RIA	NR	NR	NR	−20 °C
Roth 2007 [54]	218	Sweden	Combat	TC	122	NR	DSM-IV HTQ	AM	RIA	10%	10%	0.8 nmol/l	−70 °C
Shalev 2007 [55]	155	Israel	Road traffic accidents	TC	64	31.2 ± 11.6	DSM-IV CAPS	AM	NR	NR	NR	NR	−40 °C
Su, T 2009 [56]	27	China	Mixed trauma	NTC	2	43.15 ± 12.8	DSM-IV CAPS	AM	RIA	3%	6%	10 pg/tube	−80 °C
Wahbeh 2013 [57]	71	USA	Combat	TC	0	55.5 ± 8.9	DSM-IV CAPS	AM,PM	ELISA	4.74%	3.03%	NR	NR
Witteveen, AB 2010 [58]	1880	Netherlands	Mixed trauma	TC	141	47.0 ± 8.0	DSM-IV CAPS	AM,PM	RIA	NR	NR	NR	−20 °C
Yehuda 2005 [59]	63	USA	Holocaust	TC,NTC	36	69.7 ± 5.0	DSM-IV CAPS	AM,PM	RIA	3.90%	12.00%	10 ng/dl	NR
Yehuda, R 2005 [60]	67	USA	Holocaust	TC,NTC	34	68.5 ± 5.9	DSM-IV CAPS	AM,PM	RIA	3.90%	12.00%	10 ng/dl	NR
Young, EA 2004 [61]	516	USA	Mixed trauma	TC,NTC	457	36.8 ± 2.2	DSM-III	AM,PM	NR	6.50%	5.00%	1 ng/mL	−20 °C
Young 2004 [62]	171	USA	Mixed trauma	TC,NTC	171	18–54	DSM-III	AM,PM	NR	10%	NR	2 μg/dL	−20 °C
Steven 2004 [63]	34	USA	Childhood trauma	NTC	30	40.3 ± 3.3	DSM-IV CAPS	AM	ELISA	5.70%	6.90%	7 μg/dL	NR
Neylan 2003 [64]	32	USA	Combat	TC	0	49.4 ± 5.7	DSM-IV CAPS	AM	NR	NR	NR	NR	NR

*TC* Trauma-exposed controls, *NTC* Non-trauma-exposed controls, *RIA* Radioimmunoassay, *ELISA* Enzyme linked immunosorbent assay, *CAPS* Clinician-administered PTSD scale, *NR* Not report, *USA* United States of America, *T-frozen* Temperature of frozen

### Salivary cortisol levels in PTSD as compared with controls

**Fig. 2:**
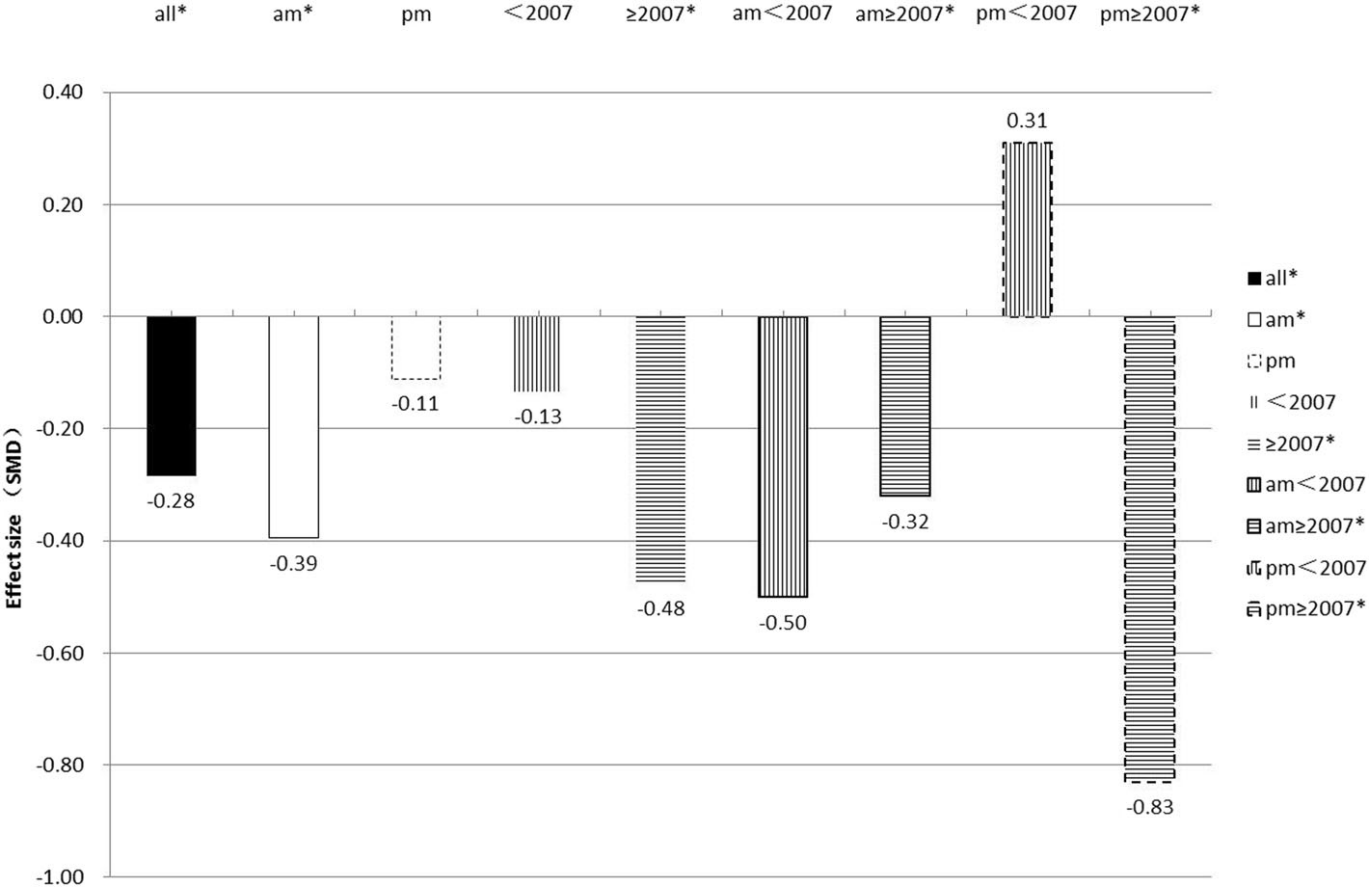
Salivary cortisol effect size. Salivary cortisol effect size (SMD) for studies examining in the morning (am), afternoon (pm), before 2007, and after 2007 levels in PTSD and control groups. PTSD,posttraumatic stress disorder; am, morning (before 12 pm); pm, afternoon (after 12 pm);<2007,before 2007;≥2007, after 2007;*p < 0.05

**Fig. 3:**
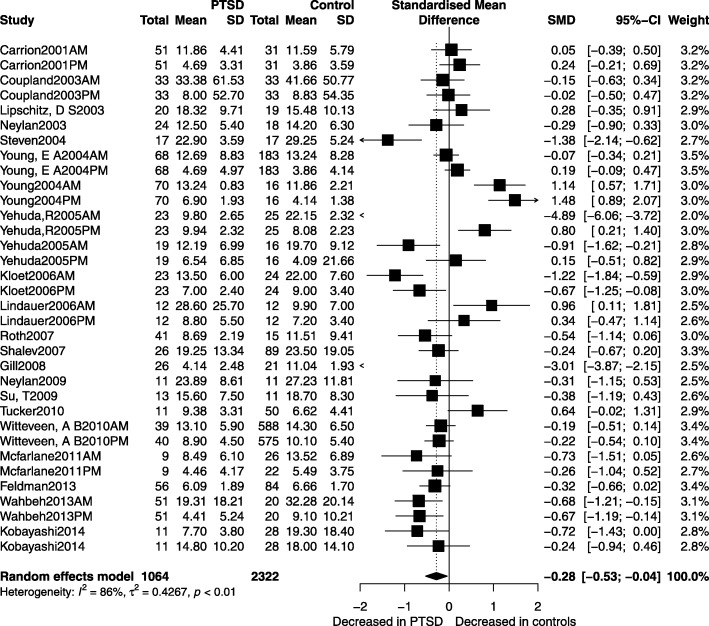
Meta-analyses of salivary cortisol

Table 2 displays the summarized salivary cortisol concentration levels between PTSD patients and the controls. A trend showing lower salivary cortisol concentration levels was observed in PTSD patients as compared to the controls (SMD = − 0.28, 95% CI -0.53; − 0.04, *p* = 0.022). There was also an overall difference in the pooled effect size between people with PTSD and NTC (SMD = − 0.33, 95% CI -0.63; − 0.03, *p* = 0.032). Figures 2 and 3 compare the salivary cortisol effect sizes (SMD) of studies using samples collected in the morning (am) with those of studies using samples collected in the afternoon (pm). The same was also compared between studies conducted before 2007 and those conducted after 2007. It should be noted that a recent similar systematic review and meta-analysis on adults was published in 2007 and it performed subgroup analysis of salivary cortisol in PTSD [12]. Since then, there has been a rapid development of various salivary cortisol analyzers. Therefore, we wanted to know whether the situation had changed 10 years later after the publication of that systematic review and meta-analysis in 2007. Hence, we performed subgroup analysis related to the eligible studies published before 2007, and those published after 2007. The results of subgroup analyses showed that the differences in salivary cortisol concentration levels between PTSD patients and the controls was bigger in studies that used samples collected in the morning than in those studies that used samples collected in the afternoon. Also, higher differences were observed in studies conducted after 2007 than in those conducted before 2007. Moreover, the biggest difference in salivary cortisol concentration level was observed in studies that used morning samples and those conducted after 2007. No differences were found for afternoon samples (*p* = 0.598), whereas in the morning people with PTSD had lower levels of cortisol than controls (SMD = − 0.39, 95% Cl − 0.70; − 0.09, *p* = 0.012). Whereas the studies conducted before 2007 did not reveal significant differences (*p* = 0.479), PTSD in studies conducted after 2007 had highly significant lower salivary cortisol levels than their comparison groups (SMD = − 0.48, 95% Cl − 0.75; − 0.20, *p* = 0.001).

**Table 2:** Meta-analysis of salivary cortisol markers in PTSD

	Participants with PTSD, *n*	controls *n*	SMD (95% CI)	*p* value	Heterogeneity			Begg’S test Kendall’s tau statistic (*p* value)
					Q statistic (DF; *p* value)	τ^2^	I^2^	
all	1064	2322	−0.28 (−0.53; −0.04)	0.022	236.00(33 < 0.0001)	0.427	86.00%	−0.1907, *p* = 0.117
am	628	1316	−0.39 (− 0.70; − 0.09)	0.012	132.68(20 < 0.0001)	0.412	84.90%	−0.2000, *p* = 0.219
pm	436	1006	−0.11 (− 0.52; 0.30)	0.598	96.40(12 < 0.0001)	0.476	87.60%	−0.1795, *p* = 0.435
<2007	659	734	−0.13 (− 0.50; 0.23)	0.479	163.59(18 < 0.0001)	0.568	89.00%	−0.2281, *p* = 0.186
≥2007	405	1588	−0.48 (− 0.75; − 0.20)	0.001	52.71(14 < 0.0001)	0.199	73.40%	−0.2952, *p* = 0.140

*PTSD* post-traumatic stress disorder, *SMD* standardised mean difference, *DF* degrees of freedom

### Meta-regression analyses

Table 3 presents the results of meta-regression analysis. It shows that country of study, sample collection time, study year, saliva cortisol assayed instrument reporting method, inter-assay variation reporting, intra-assay variation reporting, sensitivity reporting and frozen sample reporting were not significantly different. Recall that the meta-regression analysis was used to explore sources of heterogeneity among the eligible studies. If results indicate not significantly different, it means the variables in question cannot explain the overall heterogeneity. However, in our case, after introducing PTSD assessment methods into the meta-regression analysis model, results showed that sources of heterogeneity can be explained by PTSD assessment methods as the difference was significant. (b = − 0.812, 95%CI -1.540;-0.084, *p* = 0.0288).

**Table 3:** Separate univariate meta-regression model of salivary cortisol in PTSD, PTSD = post-traumatic stress disorder

	Estimate	se	zval	pval	ci.lb	ci.ub
Collection time	− 0.306	0.362	− 0.844	0.399	−1.015	0.404
Country	−0.085	0.362	−0.236	0.814	−0.795	0.624
Publication year	−0.352	0.351	−1.002	0.316	−1.041	0.337
PTSD assessment	−0.812	0.371	−2.187	0.029	−1.540	−0.084
Assayed methods	−0.344	0.384	−0.896	0.370	−1.097	0.409
Inter-assay variation	−0.090	0.391	−0.229	0.819	−0.857	0.677
Frozen	0.686	0.390	1.762	0.078	−0.077	1.450

### Heterogeneity and bias analysis

Heterogeneity was reported to be high among the eligible studies (I^2^ > 75%). However, for the subgroup analysis stratified by study year, the combined heterogeneity of studies declined from 92 to 33%. This change in heterogeneity indicated that studies conducted after 2007 had more consistent and homogeneous results.

Begg’s rank correlation test revealed no potential publication bias (*p* = 0.117), implying that there was low probability of publication bias.

## Discussion

Generally, concentrations of salivary cortisol were lower in patients with PTSD than in the controls (SMD = − 0.28, 95% CI -0.53; − 0.04, *p* = 0.022). There was also an overall difference in the pooled effect size between people with PTSD and NTC. Specifically, our findings suggest that PTSD status affects basal salivary cortisol levels; some people do not develop PTSD despite experiencing a trauma similar to that of PTSD patients [34]. The exact biological mechanisms underlying the altered long-term salivary cortisol output as a result of trauma remain largely unknown [35]. Speculatively, decreased output of salivary cortisol after developing PTSD may evolve as a compensatory anti-glucocorticoid mechanism [16, 36], to inhibit negative effects of long-term increased negative glucocorticoid feedback sensitivity of glucocorticoid receptors that have been observed in PTSD patients irrespective of trauma-exposed status. However, this remains to be further investigated [16].

As far as we know, this is the first study to perform systematic review, meta-analysis and meta-regression on the salivary cortisol concentration levels in PTSD, particularly by considering studies reporting salivary cortisol as a susceptibility marker for PTSD. Nonetheless, there are three previous systematic reviews and meta-analyses which investigated the relationship between Cortisol and post-traumatic stress disorder. One of which included only 7 salivary cortisol studies from two databases [12], another one only used 9 PTSD salivary cortisol subgroup studies [16], and the next one used only 3 salivary cortisol studies [20]. These studies found no relationship between cortisol concentration levels and PTSD. In addition, these studies did not take into account the sample source from which cortisol concentration levels were measured. For example, they included plasma/serum, saliva or urine samples, even if it is well known that the cortisol concentration levels varied from sample to sample between sufficient samples from different sources. The use of small sample sizes might have contributed to the results of no significant relationship between cortisol concentration levels and PTSD. Noteworthy, the methods for collecting salivary cortisol, testing and analyzing cortisol concentration levels have improved in more recent years. These include immunoassay or liquid chromatography-tandem mass spectrometry (LC-MS/MS) for the measurement of salivary cortisol [37, 38]. Therefore, there is need to update literature to include these new methods when investigating the relationship between cortisol concentration levels and PTSD. Accordingly, in this study, we enlarged the scope of article searching in the online electronic databases, which eventually yielded 22 eligible studies which collectively had 1064 participants with PTSD and 2322 controls. Thus, compared with the preceding previous studies, this study used relatively larger sample size which would make conclusions more comparable and convincing. Also, unlike in the preceding studies, this study focused only on salivary cortisol concentration levels when investigating its association with PTSD because results of the same investigation, using different sample sources for cortisol concentration levels, may be problematic to interpret since cortisol concentration levels differ with different sample sources. Thus, using the 22 eligible studies, some interesting significant results were found. In the analysis according to whether saliva samples were taken in the morning or in the afternoon, it was found that PTSD patients had lower levels of salivary cortisol than controls in studies which used saliva samples taken in the morning but not in those studies which used saliva samples taken in the afternoon. With regard to methodological aspects, and due to the pulsatile nature of adrenal steroid release, there are inherent limitations when using single-point measurements of basal salivary cortisol concentration levels [39]. More specifically, it is known that the cortisol release follows a circadian rhythm such as cortisol awakening response [40]. Additionally, specific basal morning salivary cortisol concentration levels seem to be lower in PTSD patients, while basal cortisol concentration level assessments at other times during the day do not seem to be associated with PTSD. Therefore, it could be speculated that PTSD patients’ cortisol awakening response was insensitive [40], whereas that of the control groups was more nimble in the morning. Considering the preceding cortisol awakening response hypothesis, it could be suggested that the ideal time for morning saliva sampling is after awakening. We suggest that researchers control for waking when saliva samples are to be collected in the morning in practical investigation (by getting people to wake at the same time, e.g. 8.00 am).

Moreover, the indicator of publication year is selected through the group discussion and the literature review, we want to evaluate that whether the previous mixed findings on cortisol levels in PTSD could be due to time of sample collection and older assays (prior to 2007). Since the recent meta-analysis article was appear in 2007 which included subgroup analysis to distribution and function of salivary cortisol in PTSD [12]. Furthermore, in the analysis according to whether studies were conducted before 2007 or after 2007, it was observed that the difference in salivary cortisol concentration levels between PTSD patients and controls was observed only in studies which were conducted after 2007. Results of Begg’s rank correlation test revealed low probability of publication bias.

In summary, this systematic review and meta-analysis found that concentrations of salivary cortisol were consistently and homogeneously lower in patients with PTSD than in the controls when the analysis included only studies conducted after 2007 and studies which used saliva samples collected in the morning. Future studies on salivary cortisol concentration levels in PTSD patients should take the following aspects into consideration:

- ① Saliva collection time should be unified. Considering the preceding cortisol awakening response hypothesis, it could be suggested that the ideal time for morning saliva sampling is after awakening. We suggest that researchers control for waking when saliva samples are to be collected in the morning in practical investigation (by getting people to wake at the same time, e.g. 8.00 am). Besides, the guidelines for assessment of the salivary cortisol must be strictly followed and these include: objective control of sampling adherence, participant instructions, sampling protocols and quantification strategies, as well as reporting and interpreting of salivary cortisol data [40].
- ② Storage methods of saliva must be unified. That is, the collected saliva sample should be stored at − 20 °C~ − 80 °C until assayed. Assay methods of saliva must be unified. We recommend using enzyme-linked immunoassay and radioimmunoassay; and inter-assay and intra-assay coefficients of variation of lower than 10%.
- ③ The meta-regression analysis had 8 variables, of which only one was significant: PTSD assessment method (Table 3). It means this variable can explain the overall heterogeneity. Thus, we recommend using DSM-IVdiagnostic criteria with the clinician-administered PTSD scale (CAPS). This scale has proved to show high degree of reliability and validity, and can provide a better reference for further study of PTSD and salivary cortisol [41]. It is expected that meeting these consensus guidelines in future research could create more powerful research designs that would yield reliable and reproducible data and results. Although no biomarkers have yet demonstrated clinical applicability for PTSD [23, 41], in future, we believe that salivary cortisol could be a quick biomarker assay to assist in screening patients for PTSD hence promising a possibility for screening a lot of people within a short time for PTSD. After screening, clinicians can then assess symptom severity by conducting clinical interviews with those suspected of having PTSD. On the one hand, as we know from natural disasters (like earthquake, hurricane, tsunami and flood) [42]; the affected population is generally large. Especially for developing countries such as China and India, with an enormous population density and limited psychiatrists, millions of people would be at high risk for PTSD after natural disasters. It can save a lot of clinicians and psychological guidance resources to enable the large-scale PTSD screening to become a reality.

Saliva is a specimen that is safe and easy to obtain in a large-scale epidemiological study. Therefore, development of rapid salivary cortisol test methods for PTSD screening could provide fast and cost-efficient means in large epidemiological studies, and in clinical practice, which could facilitate PTSD diagnosis, treatment, control and prevention.

## Conclusions

The evidence from this meta-analysis supports that salivary samples collected in the morning consistently showed a lower salivary cortisol level in patients with PTSD than in controls, although whether salivary cortisol could be used as a diagnostic tool requires further research.

## Additional file

Additional file 1: Search strategies: details of search strategy. (DOC 27 kb)

## Abbreviations

<2007: Before 2007
≥2007: After 2007
95% CI: 95% confidence interval
Am: Morning (before 12 pm)
CAPS: Clinician-administered PTSD scale
DF: Degrees of freedom
DSM-IV: Diagnostic and statistical manual of mental disorders, 4th edition
ELISA: Enzyme linked immunosorbent assay
NR: Not report
NTC: Non-trauma-exposed Controls
pm: Afternoon (after 12 pm)
PRISMA: Preferred reporting items for systematic reviews and meta-analyses
PTSD: Post-traumatic stress disorder
RIA: Radioimmunoassay
SMD: Standardised mean difference
TC: Trauma-exposed controls
