# Supplementary material for: Salivary cortisol in post-traumatic stress disorder: a systematic review and meta-analysis
Source: BMC Psychiatry. 2018 Oct 5;18:324. doi: 10.1186/s12888-018-1910-9 (PMC6173866; doi:10.1186/s12888-018-1910-9)
Supplement: Supplementary file 1 — Search strategies: details of search strategy. (DOC 27 kb) [file 12888_2018_1910_MOESM1_ESM.doc]

Search strategies: details of search strategy.

('ptsd':ab,ti OR 'posttraumatic stress disorder':ab,ti) AND ('cortisol in saliva':ab,ti OR 'saliva cortisol':ab,ti OR 'glucocorticoids in saliva':ab,ti OR 'saliva glucocorticoid':ab,ti OR 'steroid hormones in saliva':ab,ti OR 'saliva steroid':ab,ti OR 'saliva corticosteroid':ab,ti OR 'salivary cortisol':ab,ti)

153 of Embase

(((((((((((cortisol in saliva[Title/Abstract]) OR saliva cortisol[Title/Abstract]) OR glucocorticoids in saliva[Title/Abstract]) OR saliva glucocorticoid[Title/Abstract]) OR steroid hormones in saliva[Title/Abstract]) OR saliva steroid[Title/Abstract]) OR corticosteroids in saliva[Title/Abstract]) OR saliva corticosteroid[Title/Abstract])) OR Salivary Cortisol[Title/Abstract])) AND ((PTSD[Title/Abstract]) OR Posttraumatic Stress Disorder[Title/Abstract])

130 of PubMed

TS=( ptsd or post traumatic stress disorder ) AND TS= ( cortisol in saliva OR saliva cortisol OR glucocorticoids in saliva OR saliva glucocorticoid OR steroid hormones in saliva OR saliva steroid OR corticosteroids in saliva OR saliva corticosteroid OR Salivary Cortisol )

395 of Web of Science

AB ( ptsd or post traumatic stress disorder ) AND TX ( cortisol in saliva OR saliva cortisol OR glucocorticoids in saliva OR saliva glucocorticoid OR steroid hormones in saliva OR saliva steroid OR corticosteroids in saliva OR saliva corticosteroid OR Salivary Cortisol )

106 of PsycARTICLES
